# Spontaneous Anterolateral Papillary Muscle Rupture Complicated by Acute Torrential Mitral Regurgitation and Cardiogenic Shock: A Case Report

**DOI:** 10.7759/cureus.84304

**Published:** 2025-05-17

**Authors:** Karuna Rayamajhi, Fnu Parul, Rohan Kumar, Mahmoud Khairy, Michael J Kehdi

**Affiliations:** 1 Internal Medicine, University of Michigan/Sparrow Hospital, Lansing, USA; 2 Medicine, Michigan State University, Lansing, USA; 3 Cardiology, University of Michigan/Sparrow Hospital, Lansing, USA

**Keywords:** anterolateral papillary muscle, assist device, cardiogenic shock, mitral regurgitation, papillary muscle rupture

## Abstract

Spontaneous papillary muscle rupture is a rare and life-threatening event. We present the case of a 70-year-old male who presented with shortness of breath and cough, with imaging findings consistent with flash bilateral pulmonary edema. Due to progressive respiratory failure and cardiogenic shock, he required emergent intubation and initiation of inotropic and vasopressor support. Transthoracic echocardiogram was concerning for a flail anterior mitral leaflet and showed normal left ventricular systolic function. As there was concern for acute mitral regurgitation, an emergent transesophageal echocardiography was performed and showed severe mitral regurgitation with a flail anterior mitral leaflet secondary to a ruptured anterolateral papillary muscle. The patient underwent emergent intra-aortic balloon pump placement and coronary angiography, which revealed 80% stenosis of the proximal left anterior descending artery. Subsequently, the patient underwent mitral valve replacement and single-vessel coronary artery bypass grafting (CABG) with the initiation of veno-arterial extracorporeal membrane oxygenation (VA-ECMO). Post-surgery, a repeat transthoracic echocardiography (TTE) revealed severe biventricular systolic dysfunction. His hospital course was further complicated by the development of a large left atrial thrombus, necessitating removal of VA-ECMO and subsequently placement of an Impella (Abiomed, Danvers, MA) and right ventricular assist device (RVAD). After a prolonged hospital stay, the patient’s condition stabilized, and he was discharged home with follow-up echocardiography demonstrating a recovered ejection fraction.

## Introduction

Papillary muscle rupture (PMR) is a rare, life-threatening condition that can occur in both ischemic and non-ischemic settings, including infective endocarditis, trauma, and myocarditis [1]. Following an acute myocardial infarction, PMR occurs in approximately 0.07% to 0.26% of cases and accounts for 5% of post-myocardial infarction mortality [2]. Spontaneous PMR is exceptionally uncommon, with only a few reported cases, and is associated with high in-hospital mortality due to the resulting severe hemodynamic instability. Without surgical intervention, mortality approaches 50% within 24 hours, making urgent surgical repair the cornerstone of management [2]. Here, we present a case of spontaneous PMR leading to severe acute mitral regurgitation (MR) and cardiogenic shock. This case is clinically significant, as it highlights the need for a high index of suspicion, especially in patients who do not present with signs of acute myocardial ischemia, yet follow the same fatally complicated course with rapid hemodynamic collapse as seen in typical cases of acute PMR.

## Case presentation

**Figure 1:**
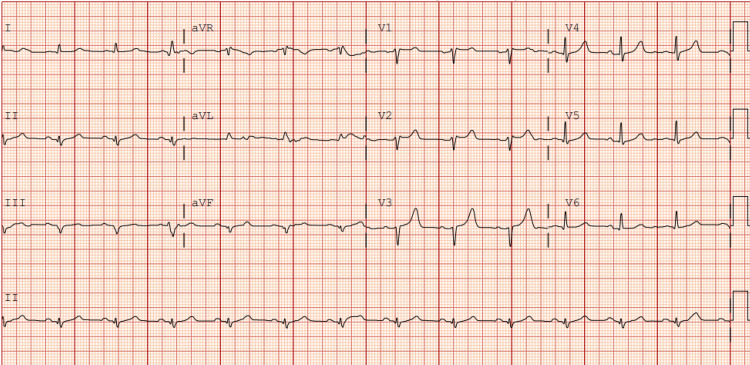
EKG showing incomplete right bundle branch block with left axis deviation

**Figure 2:**
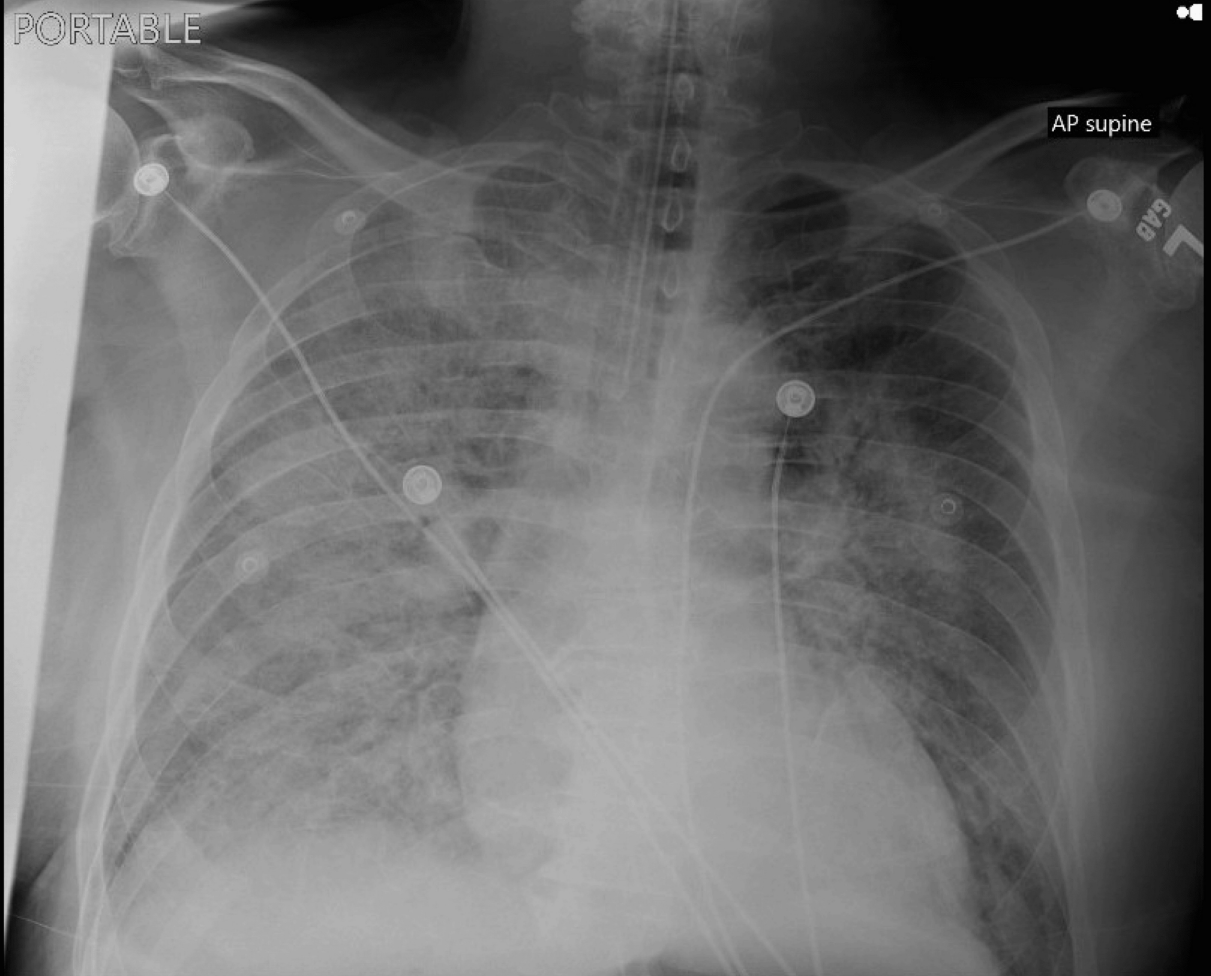
Chest X-ray showing bilateral pulmonary edema, right more than left

A 70-year-old male with a history of chronic smoking but no other significant medical conditions presented with acute shortness of breath, cough, and pink frothy sputum that began a day prior. He denied chest pain, trauma, or constitutional symptoms. On arrival, he was tachycardic (122 beats/minute), tachypneic (27 breaths/minute), and hypoxic (SpO₂ 85%), requiring high-flow nasal cannula oxygen support. Physical examination revealed bilateral posterior lung crackles and a loud holosystolic murmur at the cardiac apex. ECG showed sinus tachycardia with incomplete right bundle branch block, left axis deviation, and nonspecific ST-wave changes (Figure 1). Laboratory findings were notable for elevated high-sensitivity troponin, D-dimer, B-type natriuretic peptide (BNP), and lactate, representing myocardial injury with signs of volume overload and hypoperfusion (Table 1). Chest radiography showed bilateral infiltrates and effusions, more pronounced on the right, suggestive of pulmonary edema (Figure 2). At this point, differential diagnoses include acute systolic heart failure exacerbation, acute respiratory distress syndrome (ARDS), severe pneumonia, and acute MR supported by unilateral pulmonary edema.

**Table 1:** Lab findings on admission BNP - B-type natriuretic peptide

Labs	Value	Reference
High sensitivity troponins	2724 ng/L	0-8 ng/L
D-dimer	2.87 mg/L	0.00-0.5 mg/L
BNP	192 pg/mL	0-100 pg/mL
Lactate	8.2 mmol/L	0.2-1.8 ml/L

**Figure 3:**
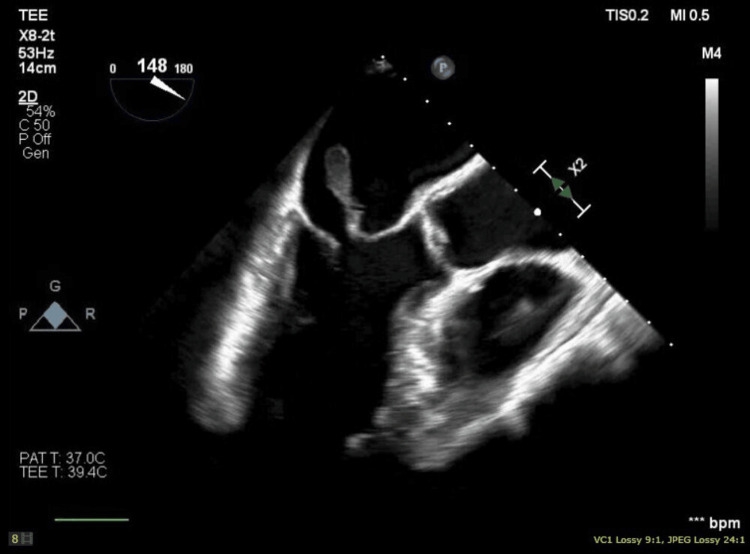
Transesophageal echocardiogram mid-esophageal long-axis view showing the flail papillary muscle

**Figure 4:**
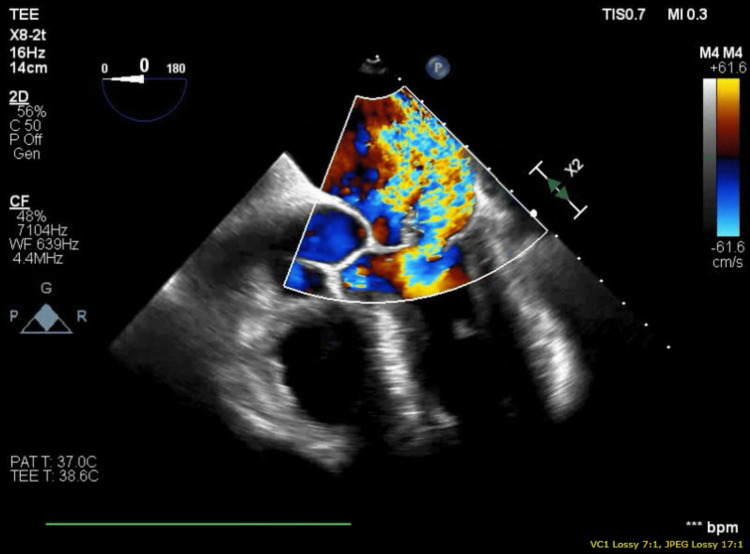
Transesophageal echocardiogram mid-esophageal five-chamber view showing severe mitral regurgitation

**Figure 5:**
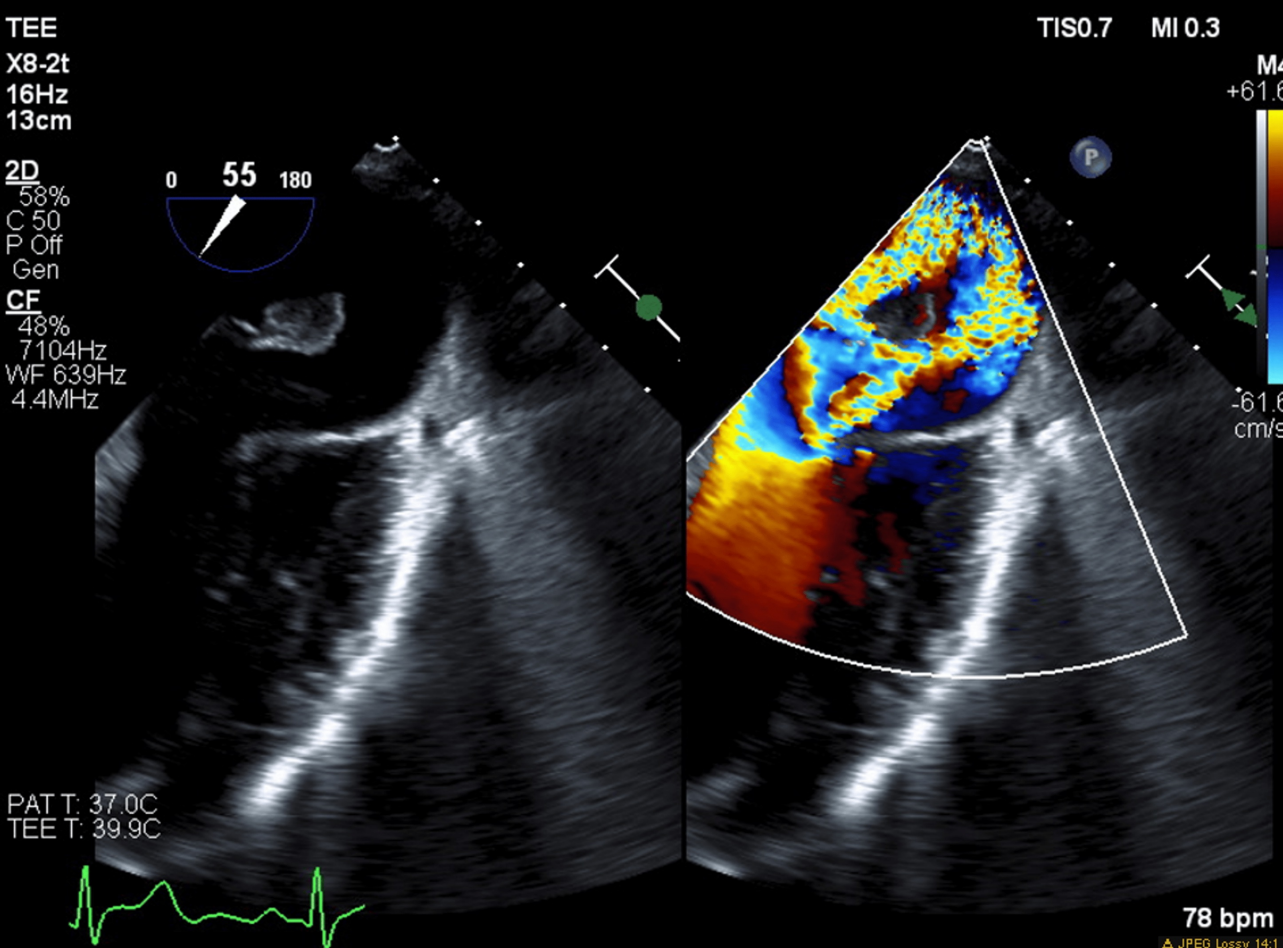
Transesophageal echocardiogram midesophageal two-chamber view showing flail papillary muscle and severe mitral regurgitation

The patient further acutely deteriorated, necessitating emergent intubation and inotropic and vasopressor support with subsequent admission to the intensive care unit (ICU). Urgent transthoracic echocardiography (TTE) revealed severe MR with a flail anterior leaflet and preserved ejection fraction (55-60%). Further, transesophageal echocardiography (TEE) confirmed acute severe MR with a ruptured anterolateral papillary muscle resulting in a flail anterior mitral leaflet prolapsing into the left atrium (Figures 3-5). An emergent left heart catheterization was done along with intra-aortic balloon pump (IABP) placement within two to four hours of admission. Coronary angiography revealed 80% proximal stenosis of the left anterior descending (LAD) artery and 20% stenosis in the left circumflex (LCX) and right coronary artery. The patient underwent emergent mitral valve replacement (MVR) with a 27 mm bioprosthetic mitral valve and coronary artery bypass grafting (CABG) (left internal mammary artery (LIMA) to LAD), along with the initiation of preoperatively planned veno-arterial extracorporeal membrane oxygenation (VA-ECMO) due to worsening end-organ damage, including acute kidney injury and rising lactate levels within 12 hours of admission. Intraoperative TEE revealed severe left ventricular systolic dysfunction, which was a postoperative development.

He was transferred to a higher center for further management, as our center was not ECMO capable, where repeat TTE demonstrated severe biventricular systolic dysfunction. On day 4 of being on VA-ECMO, the patient developed a large left atrial thrombus, necessitating the removal of VA-ECMO and implantation of an ascending aortic Impella (Abiomed, Danvers, MA) and right ventricular assist device (RVAD). His hemodynamics improved after a prolonged ICU stay, allowing for the removal of Impella and RVAD on day 17th of the hospitalization. 

Pathologic examination of the excised anterior mitral leaflet and papillary muscle showed myxoid degeneration of the valve tissue, along with benign cardiac muscle and fibrinous material, without any evidence of necrosis. He was discharged after 45 days of hospital stay on aspirin, clopidogrel, statin, and a beta-blocker, with a referral to cardiac rehabilitation. At one-month follow-up, TTE showed recovered biventricular systolic function (EF 60%) with a functional bioprosthetic valve and no valvular or paravalvular leak. 

## Discussion

Papillary muscles play a crucial role in the function of the mitral valve by anchoring the valve leaflets via the chordae tendineae, thereby preventing prolapse of the valve into the left atrium during systole. The two papillary muscles in the left ventricle are the anterolateral and posteromedial muscles, each with distinct blood supplies. 

PMR is a rare, life-threatening cardiac emergency most commonly associated with acute myocardial infarction. It carries a high mortality risk and requires emergent surgical intervention. Nonischemic, spontaneous PMR is even rarer and has been linked to conditions such as Ehlers-Danlos syndrome, myocarditis, trauma, and mitral annular calcification. However, no clear etiology can be identified in some cases, including ours. A review of nonischemic spontaneous PMR cases identified only 11 additional cases over a 15-year period, highlighting its rarity [1]. Rupture of the anterolateral muscle is uncommon due to its dual blood supply from the LAD artery and the diagonal or marginal branch of the circumflex artery, compared to the posteromedial papillary muscle, which has a single blood supply from the circumflex or right coronary artery, depending on coronary dominance [1]. This finding supports the hypothesis that microvascular ischemia may contribute to PMR. In the presented case, while the etiology of the anterolateral PMR remains somewhat ambiguous, particularly in the context of an 80% LAD occlusion, the presence of a patent LCX and histopathological findings showing myxoid degeneration without evidence of necrosis suggest a predominantly non-ischemic etiology. At most, the rupture may be considered multifactorial, likely involving age-related or idiopathic degenerative changes and microvascular ischemia rather than a direct consequence of significant epicardial coronary artery disease.

Regardless of the underlying cause, acute MR resulting from PMR leads to a rapid rise in left atrial and pulmonary pressures, often causing pulmonary edema and cardiogenic shock. Without prompt intervention, rupture carries an estimated 50% mortality risk within 24 hours [2]. Surgical correction is, therefore, essential, with MVR being the preferred approach in most cases. The American College of Cardiology and American Heart Association (ACC/AHA) guidelines recommend MVR over mitral valve repair in this setting, as it is associated with better survival and ventricular function despite a perioperative mortality rate of approximately 20% [3]. However, mitral valve repair may be considered in select cases, mainly when the rupture is partial or when the valve apparatus can be preserved. The American Association for Thoracic Surgery (AATS) suggests that repair using a small, rigid annuloplasty ring can be effective, though the risk of recurrent MR remains high. Subvalvular repair techniques, such as papillary muscle approximation, have shown promise in improving left ventricular geometry and reducing MR recurrence when combined with restrictive annuloplasty [4]. Furthermore, performing CABG for concomitant coronary artery disease is advised. Studies have shown that CABG, in conjunction with mitral valve surgery, can confer a survival benefit [5]. Postoperative outcomes depend on hemodynamic stability and the timeliness of intervention. Advances in perioperative support, including IABPs, VA-ECMO, and ventricular assist devices (VADs), have improved survival in critically ill patients undergoing surgery for PMR. In our case, VA-ECMO was crucial in stabilizing the patient, facilitating recovery, and ultimately allowing for the removal of mechanical circulatory support. Long-term prognosis is generally favorable if patients survive the acute phase, with many achieving normalization of left ventricular function post-surgery. However, careful postoperative monitoring, including yearly serial echocardiograms, is essential to detect complications such as valve dysfunction or thrombosis and to ensure optimal cardiac recovery. 

## Conclusions

Spontaneous rupture of the papillary muscle is a rare but life-threatening condition that typically results in acute, severe - often torrential - MR, leading to rapid hemodynamic deterioration and cardiogenic shock. In cases of spontaneous PMR, including the one described here, diagnostic ambiguity often persists, underscoring the need for a high index of clinical suspicion. Histopathological examination of the excised tissue, in conjunction with clinical presentation, can help differentiate ischemic from non-ischemic etiologies. Notably, anterolateral muscle rupture is less common than posteromedial rupture. Without prompt surgical intervention, mortality remains exceedingly high. Thus, early diagnosis, timely mechanical support, and emergent MVR are essential to improving patient outcomes.
